# Refining the Choosing Health Infant feeding for Infant Health intervention and implementation strategy: Re-CHErIsH Study Protocol

**DOI:** 10.12688/hrbopenres.13935.1

**Published:** 2024-09-26

**Authors:** Eibhlín Looney, Moira Duffy, Helen Ahern Galvin, Molly Byrne, Rebecca Golley, Catherine Hayes, Tony Heffernan, Aisling Jennings, Brittany Johnson, Patricia M Kearney, Colette Kelly, Patricia Leahy-Warren, Marian McBride, Sheena McHugh, Kate O’Neill, Sarah Redsell, Anna Lene Seidler, Elaine Toomey, Karen Matvienko-Sikar

**Affiliations:** 1 School of Public Health, University College Cork, Cork, County Cork, Ireland; 2 RPHN, North Cork Community Care Area, Heath Service Executive (HSE) South, County Cork, Ireland; 3 School of Psychology, University of Galway, Galway, County Galway, Ireland; 4 College of Nursing and Health Sciences, Flinders University, Adelaide, South Australia, Australia; 5 Public Health and Primary Care, School of Medicine, The University of Dublin Trinity College, Dublin, Leinster, Ireland; 6 Mallow Primary Healthcare Centre, Cork, County Cork, Ireland; 7 Department of General Practice, University College Cork, Cork, County Cork, Ireland; 8 School of Health Sciences, University of Galway, Galway, County Galway, Ireland; 9 School of Nursing and Midwifery, University College Cork, Cork, County Cork, Ireland; 10 Health & Wellbeing, Strategy & Research, Healthcare Strategy, Health Service Executive, County Dublin, Ireland; 11 School of Health Sciences, University of Nottingham, Nottingham, England, UK; 12 National Health and Medical Research Council Clinical Trials Centre, The University of Sydney, Sydney, New South Wales, Australia; 13 School of Nursing and Midwifery/Centre for Health Evaluation, Methodology Research and Evidence Synthesis, University of Galway, Galway, County Galway, Ireland

**Keywords:** Childhood obesity, infant feeding, primary care, intervention

## Abstract

**Background:**

Childhood obesity is a significant global public health challenge, with significant adverse effects on both mental and physical health outcomes. During the period from birth to one-year, modifiable caregiver behaviours, such as what, how and when infants are fed, can influence obesity development and prevention. The Choosing Healthy Eating for Infant Health (CHErIsH) intervention was developed to support healthy infant feeding practices to prevent childhood obesity in the first year. A feasibility study examined acceptability and feasibility of the CHErIsH intervention in primary care and identified key challenges and possible areas for refinement of the intervention and trial processes. The current project aims to refine delivery of the CHErIsH intervention and trial processes to maximise the likelihood of successful future implementation and evaluation.

**Methods:**

This study will utilise a mixed-methods approach and will be conducted in three phases. In Phase 1 potential refinements to the CHErIsH intervention delivery and trial processes will be developed from a review of the feasibility study findings and input from the multidisciplinary team. An online mixed-methods survey will be conducted in Phase 2 to evaluate caregiver attitudes about the proposed refinements from Phase 1. Participants will be pregnant women, their partners, and/or parents/primary caregivers of infants up to 2-years of age, based in Ireland. Participants will be recruited using convenience and snowball sampling. In Phase 3 a stakeholder consensus meeting, using the nominal group technique, will be conducted to agree the refined intervention and trial processes. Stakeholders will include healthcare professionals, researchers, policymakers, and parents/caregivers, who will discuss and rate refinements in terms of preference.

**Conclusions:**

Findings from this study will address uncertainties in the intervention delivery and trial processes of the CHErIsH intervention, with the potential to maximise the likelihood of successful future implementation and evaluation of a primary-care based obesity prevention intervention.

## Introduction

Childhood obesity is a significant global public health challenge. Approximately 37 million children under the age of 5 years are living with overweight or obesity ( World Health Organisation, 2024). Childhood obesity can have significant adverse effects on mental and physical health outcomes both in childhood and into later life. These include an increased risk of developing joint problems ( Molina-Garcia *et al.*, 2021), hypertension ( Reilly & Kelly, 2011), type 2 diabetes ( Bacha & Gidding, 2016; Horesh *et al.*, 2021; Reilly & Kelly, 2011), coronary heart disease, and stroke ( Drozdz *et al.*, 2021; Horesh *et al.*, 2021; Reilly & Kelly, 2011). Those living with childhood obesity are at heightened risk of experiencing depression, anxiety, and poorer self-esteem, compared with their healthy-weight peers ( Harriger & Thompson, 2012; Rankin *et al.*, 2016). Childhood obesity is also associated with long-term morbidity and pre-mature mortality ( Horesh *et al.*, 2021; Maffeis & Tatò, 2001; Reilly & Kelly, 2011) and lower health-related quality of life ( Killedar *et al.*, 2020; Rankin *et al.*, 2016).

The period from birth to one year is critical for the development and prevention of childhood obesity ( Blake-Lamb *et al.*, 2016; Woo Baidal *et al.*, 2016). During this period, obesity development can be influenced by modifiable factors such as infant feeding behaviours ( Clark *et al.*, 2007; Woo Baidal *et al.*, 2016). Infant feeding involves parent/caregiver behaviours related to what, how and when infants are fed. This includes factors related to the initiation and duration of breastfeeding ( Patro-Gołąb *et al.*, 2016; Pluymen *et al.*, 2018), the timing of the introduction of solids ( Papoutsou *et al.*, 2018; Pluymen *et al.*, 2018; Wang *et al.*, 2016), the types of complementary foods fed to children ( Pearce & Langley-Evans, 2013), and caregiver–child feeding interactions such as responsiveness to hunger and satiety cues ( Birch & Doub, 2014; Hurley *et al.*, 2011).

As the period from birth to one year is an important time to establish healthy eating habits, there is an increased focus on interventions during this period to prevent childhood obesity ( Blake-Lamb *et al.*, 2016; Hennessy *et al.*, 2019; Koplin *et al.*, 2019; Redsell *et al.*, 2016). Healthcare professionals (HCPs), particularly those in primary care such as general practitioners (GPs), practice nurses, (PNs), as well as public health nurses (PHNs), are well placed to support and inform parents and caregivers around early infant feeding and to deliver infant feeding and childhood obesity prevention interventions. One such intervention is the Choosing Healthy Eating for Infant Health (CHErIsH) intervention, which was developed to support healthy infant feeding practices to prevent childhood obesity in Ireland ( Toomey *et al.*, 2020). The intervention involves brief, consistent, verbal messages about infant feeding (breastfeeding and complementary feeding) for parents and caregivers, along with the provision of supporting materials (infant bib, magnet, and information leaflet) on infant feeding. The CHErIsH intervention was developed to be delivered in primary care in Ireland by HCPs at the childhood vaccination visits (infant age 2, 4, 6, 12 and 13 months). To support intervention delivery, a HCP-level implementation strategy was also developed which included 1) a local opinion leader, 2) incentivised training and supports for HCPs, 3) distribution of supporting resources and education materials to HCPs, 4) electronic delivery prompts during the vaccination visit, and 5) awareness raising across HCPs within the clinical practice and local primary care community ( Toomey *et al.*, 2020). The intervention, HCP-level implementation strategy and their underpinning theory were developed following a robust process involving integrated multidisciplinary stakeholder engagement ( Toomey *et al.*, 2020).

A feasibility study previously examined the acceptability and feasibility of delivery of the CHErIsH intervention and HCP-level implementation strategy in a primary care centre in the south of Ireland ( Matvienko-Sikar *et al.*, 2019). Unpublished findings from the focus groups with HCPs following the feasibility study demonstrated increased HCP knowledge and positive attitudes about infant feeding. HCPs involved in the intervention and implementation strategy also reported that training, support and supporting materials were useful and fit for purpose. However, key challenges and areas for refinement were identified relating to trial processes and delivery of the intervention. For instance, recruitment of participants was very low (n=3), which meant that feasibility of the intervention and trial processes could not be evaluated. In addition, HCPs reported that the intervention was not always delivered during vaccination visits, as the vaccination was the primary function of this visit. These findings highlight the valuable potential of the CHErIsH intervention, but the need to refine intervention delivery and recruitment processes to enable more comprehensive and robust evaluation of the impact of the intervention on infant feeding and childhood obesity outcomes.

### Aims

The aim of this project is to refine recruitment processes and delivery of the CHErIsH intervention to maximise the likelihood of successful future implementation and evaluation. In doing so the proposed project will address key uncertainties about CHErIsH recruitment processes and intervention delivery.

## Methods

This study will utilise a mixed-methods approach and will be conducted in three phases. The study is ongoing, Phase 1 of the study has been completed.

### Phase 1: Development of potential refinements to the CHErIsH recruitment processes and intervention

The objective of Phase 1 is to develop potential refinements to the recruitment processes and intervention delivery of the CHErIsH intervention. Preliminary unpublished findings from the CHErIsH feasibility study ( Matvienko-Sikar *et al.*, 2019) will be reviewed and examined by EL, MD and KMS, to identify, clarify and further develop potential refinements to the intervention and the trial processes. Refinements developed from that review will then be circulated to the multidisciplinary team who will provide input on the potential improvements to the recruitment processes and intervention delivery. This multidisciplinary team includes international experts in childhood obesity, infant feeding, dietetics, general practice, nursing and midwifery, implementation science, health psychology, health promotion, health policy, and health behaviour change. The multidisciplinary team will provide two rounds of written feedback on the proposed refinements, which will be incorporated to finalise a list of potential refinements. Potential refinements agreed by the project team will be used in Phase 2 and Phase 3.

### Phase 2: Online survey to evaluate caregiver attitudes and perspectives about proposed refinements

The objective of Phase 2 is to evaluate caregiver attitudes and perspectives about the proposed refinements developed in Phase 1 using a mixed-methods survey.

***Participants and recruitment.*** Participants in the survey will be pregnant women, their partners, and/or parents/primary caregivers of infants up to 2 years of age.

Inclusion criteria:

- Being a pregnant woman, a partner of a pregnant woman, and/or a parent/primary caregiver of a child up to two years old in Ireland.
- ≥ 18 years of age and can be biological or non-biological parents.
- Resident in Ireland from pregnancy to the time of survey completion.
- Based anywhere in Ireland and not excluded based on any sociodemographic factors, such as ethnicity, socioeconomic status.

Exclusion criteria:

- People who are not pregnant, or the partner of a pregnant woman; or who only have children over two years of age.
- People not living in Ireland.
- People younger than 18 years of age.

Convenience and snowball sampling strategies will be used to recruit participants. Pregnant women, their partners, and parents/primary caregivers will be recruited via social media (e.g. Instagram, X (formerly Twitter), TikTok, Facebook, Reddit), pregnancy and/or parenting websites and online forums (including feedings and parenting groups). Study recruitment information (please see Extended Data) will be posted on these forums, dependent on approval of forum administrators. Recruitment will focus on obtaining a diverse, representative sample including those experiencing socioeconomic disadvantage and minority groups. All recruitment materials will include a QR code for ease of access to study information online, and all materials will be written in lay-language. As this is an exploratory descriptive survey, sample size calculations have not been conducted. This study will aim to recruit as many participants as possible, with a minimum sample size of 400 participants. This number is feasible based on previous recent survey research ( Looney *et al.*, 2024b) conducted with this population.

***Data collection.*** A mixed-methods survey will be developed including both closed and open-ended questions and will be informed by the Theoretical Framework of Acceptability (TFA) ( Sekhon *et al.*, 2017) (please see Extended Data for full study questionnaire). Closed-ended questions will include demographic information and participant ratings of proposed recruitment and intervention delivery refinements. Proposed recruitment and intervention refinements will be rated by participants in terms of affective attitude, burden, ethicality, perceived effectiveness, and overall preference where multiple potential refinements exist. A 5-point Likert scale from “strongly disagree” to “strongly agree” will be used for participant ratings of proposed refinements. Open-ended questions will ask participants their thoughts and further comments about the refinements. Data collection will take place online using Qualtrics.

***Data analysis.*** A summary score for each question will be generated ( Sekhon *et al.*, 2022). Summary scores, as well as the individual item scores, will be analysed descriptively to determine participant-preferred refinements. Open-ended questions will be analysed using reflexive thematic analysis ( Braun & Clarke, 2021). Quantitative and qualitative data will be triangulated to inform iterations to proposed refinements and will be used in Phase 3.

### Phase 3: Stakeholder consensus meeting to determine and agree refined trial processes and the intervention

The objective of Phase 3 is to determine and agree refined trial processes and the intervention with stakeholders. This will be completed via a stakeholder consensus meeting.

***Participants and recruitment.*** The consensus meeting component of this study will include approximately 15 stakeholders who are experts in the fields of infant feeding and childhood obesity based on their profession and/or their experience. These stakeholders will include healthcare professionals (e.g., general practitioners, practice nurses, midwives, public health nurses, dietitians), researchers, policymakers, and parents/caregivers. Stakeholders will be invited to the meeting via professional organisations (e.g., the Irish College of General Practitioners), existing contacts, and social media. Written and/or email invitations shall also be distributed to, for example, Directors of Public Health Nursing nationwide and parenting and pregnancy groups in order to invite participation from a wide range of stakeholders.

***Data collection.*** The stakeholder consensus meeting will be held using Microsoft Teams. A nominal group technique approach will be used ( McMillan *et al.*, 2016). Proposed refinements to the recruitment processes and intervention delivery, informed by Phase 1 and Phase 2, will be presented to stakeholders. Stakeholders will engage in facilitated breakout group discussions about the refinements, structured around the TFA components and allowing for additional stakeholder-proposed refinements. Stakeholders will reconvene for a full group discussion, after which they will rate refinements in terms of preference, keeping TFA components in mind. Rating will be followed by further discussion as needed, and a final round of rating. Stakeholders will rate refinements in terms of preference using the free version of Mentimeter software ( https://www.mentimeter.com/). The stakeholder consensus meeting will be recorded using Microsoft Teams, with automatic transcription also via Microsoft Teams. The transcript will be reviewed for accuracy against the recording and anonymised, after which the recording will be deleted. At the end of the meeting participants will be reminded of the aims of the study, what will happen with the data they provided, and what the next steps of the project are.

***Data analysis.*** Stakeholder ratings will be collected during the meeting. The ratings will be descriptively analysed to determine the most appropriate and acceptable refinements to include in the recruitment processes and intervention delivery. The qualitative transcript will be analysed using framework analysis, using the TFA as the analytic framework.

### Public and patient involvement (PPI)

Public and patient involvement (PPI) will be embedded in the study in three ways; through input from PPI representatives during the study; through the stakeholder consensus meeting in Phase 3; and through the input of the multidisciplinary research team. PPI representatives will comprise of a pregnant woman, and a mother of two young children (one under 1 year and one under 2 years of age). Both representatives are Irish and currently living in Ireland. The two PPI representatives will be involved in the study in Phase 2 and Phase 3. PPI representatives reviewed the study materials, including the online survey to evaluate caregiver attitudes and perspectives about proposed refinements in Phase 2. Following review of study materials, the PPI representatives met with a member of the research team (KMS) to discuss and improve the materials. As a result of PPI involvement survey questions were changed, clarified, and improved. PPI will also be included in Phase 3 through the determination and agreement of refined trial and intervention processes via a stakeholder consensus meeting. This will involve engagement with diverse stakeholders including health care professionals, Health Service Executive representatives, and parents/caregivers. The involvement of PPI representatives in the co-development of the refinements will ensure that the refinements are appropriate and acceptable to these stakeholders. The multidisciplinary research team will provide input at all stages of the study. This multidisciplinary team includes health care professionals (general practitioners, midwifery, and public health nursing) as well as international experts in childhood obesity, infant feeding, dietetics, implementation science, health psychology, health promotion, health policy, and health behaviour change.

## Dissemination

This study will be registered on Open Science Framework. The accompanying data and materials will also be made openly available upon study completion on the Open Science Framework. Findings from this study will be disseminated in a peer-reviewed publication, and as a presentation at relevant conferences, seminars, and research meetings. An infographic and plain language summary for parents and caregivers will also be developed and shared via social media and online.

## Discussion

The aim of this project is to address key uncertainties in the recruitment processes and intervention delivery of the CHErIsH intervention through refining, evaluating, and agreeing on updates to these trial systems. This will maximise the likelihood of successful future implementation and evaluation of the CHErIsH intervention. The refined recruitment processes and intervention delivery resulting from this project can be tested in future pilot and definitive trials with potential to optimise infant feeding and prevent childhood obesity outcomes.

## Ethics and consent

All research activities will be conducted following the University College Cork (UCC) Code of Research Conduct, ethical approval, and in accordance with General Data Protection Regulations (GDPR). Ethical approval will be obtained prior to commencement of Phase 2 from the University College Cork Social Research Ethics Committee. The study is currently being reviewed by the University College Cork Social Research Ethics Committee and Phase 2 will not commence until ethical approval is obtained. Participants (Phase 2) and stakeholders (Phase 3) will be provided with full study information prior to completing the survey (please see Extended Data) and stakeholder consensus meeting (please see Extended Data) respectively. The information leaflets will detail the aims of the current research, what is involved in participating, and their ethical entitlements, along with research team contact information. The study information will make clear that participation is voluntary. Survey participants can withdraw from the study at any stage up to the point of online submission of survey responses, at which point it will not be possible to identify individual survey submissions due to their anonymous nature. Stakeholders can choose to withdraw at any time during the stakeholder consensus meeting. Participants and stakeholders will also be informed of data management and protection within the study, and how their data will be used. Informed consent will be obtained online as electronic written consent prior to commencement of the online survey and stakeholder consensus meeting (please see Extended Data). At the end of the online survey, the study aims, and information will be restated, including information about what will happen to participant’s submitted data. Further, participants will be provided with contact details for appropriate supports and resources, should they experience any distress. These resources will include contact details for, for example, the Specialist Perinatal Mental Health Services in Cork University Maternity Hospital and reference to their local healthcare providers. A link will also be provided to the MyChild.ie website for resources and information concerning infant feeding and other child health topics.

Quantitative data in the form of participant rankings and qualitative data in the form of transcribed discussions will be recorded and stored anonymously. The aggregated anonymous dataset will be made available following completion of the study in line with best open science practices and the Findability, Accessibility, Interoperability, and Reuse of digital assets (FAIR) Principles. Information about sharing of the dataset is included in the participant information leaflet and consent form. All data will be stored in accordance with the European GDPR and the UCC data protection policy, and the FAIR Principles. Data will be stored for a minimum of ten years for audit purposes as per the UCC code of Research Conduct and the Data Protection Act. All data will be stored securely on the UCC- supplied OneDrive for Business using Microsoft Teams.

## Data Availability

No data are associated with this article. Open Science Framework: Re-CHErIsH. https://doi.org/10.17605/OSF.IO/V2XB6 ( Looney *et al.*, 2024a). List of contents in repository: GUIDED Checklist_Complete.pdf Re-CHErIsH Survey_Final.docx Stakeholder Consent Form.docx Stakeholder Email Invitation.docx Stakeholder Information Leaflet.docx Survey Participant Consent Form.docx Survey Participant Information Leaflet.docx Survey Recruitment Poster.docx Data are available under the terms of the Creative Commons Attribution 4.0 International license (CC-BY 4.0).
