# Supplementary material for: Refining the Choosing Health Infant feeding for Infant Health intervention and implementation strategy: Re-CHErIsH Study Protocol
Source: HRB Open Res. 2024 Sep 26;7:59. [Version 1] doi: 10.12688/hrbopenres.13935.1 (PMC11808839; doi:10.12688/hrbopenres.13935.1)
Supplement: Peer review history

# Reviewer report: Reviewer response for version 1

**DOI:** 10.21956/hrbopenres.15290.r43446

**Published:** 2024-12-17

**Authors:** Michelle W Katzow

**Affiliations:** 1 Department of Pediatrics, Northwell Health, Glen Cove, New York, USA

This paper presents the proposed protocol for a mixed methods study to refine the CHErIsH intervention, an early child obesity prevention program implemented in primary care settings in Ireland from birth to age 1 year. This is an important topic for research in obesity prevention and the field will certainly benefit from transparent intervention development processes and plans. Overall, the paper would benefit from a bit more detail regarding the initial feasibility study’s failure and justification for the procedures proposed.

 Introduction

1. The sample size of 3 for the feasibility study should be explained further Was this 3 primary care patients or 3 health care providers?.
2. Is there an existing process or framework that the authors are using to guide this intervention development study in three phases? How were the components of these phases decided upon? Is there evidence that that this particular process for refining an intervention will result in improvements?  
3. It would be helpful to understand more details about the recruitment processes and intervention delivery that are being refined.

Methods

1. Phase 1 is said to be complete but then described in the future tense, which is confusing. I recommend changing this paragraph to the past tense if it truly has all already occurred.
2. If Phase 1 is complete, then the results of phase 1 should be presented, as they inform the details of Phase 2. The reader can then understand plans for Phase 2 in more detail.
3. Inclusion and exclusion criteria should be revised to more clearly delineate what the actual criteria are. “Not excluded based on any sociodemographic factors, such as ethnicity, socioeconomic status” is not helpful without listing the factors themselves. Additionally, if these are exclusion criteria, they should be listed under exclusion criteria instead of the absence of the 3 inclusion criteria. Are there any other factors that may apply to someone who otherwise meets inclusion criteria but might not be appropriate for the study? Severe medical or psychiatric illness? Known congenital anomaly that will interfere with feeding?
4. Are there specific plans to reach “experiencing socioeconomic disadvantage and minority groups.”
5. What is the justification for a sample of at least 400? If the goals of the study can be achieved with 200, then it is not appropriate to survey 400, even if the study is exploratory and descriptive.
6. What is the nominal group technique approach? Is it the approach that is laid out in the data collection paragraph? Or are those procedures separate from the nominal group technique approach?

Is the study design appropriate for the research question?

Partly

Is the rationale for, and objectives of, the study clearly described?

Partly

Are sufficient details of the methods provided to allow replication by others?

Partly

Are the datasets clearly presented in a useable and accessible format?

Not applicable

Reviewer Expertise:

Early childhood obesity prevention beginning in pregnancy, including iterative intervention development.

I confirm that I have read this submission and believe that I have an appropriate level of expertise to confirm that it is of an acceptable scientific standard, however I have significant reservations, as outlined above.

# Reviewer report: Reviewer response for version 1

**DOI:** 10.21956/hrbopenres.15290.r43080

**Published:** 2024-12-13

**Authors:** Ana Paola Campos

**Affiliations:** 1 The University of North Carolina at Chapel Hill, Chapel Hill, North Carolina, USA

This paper briefly describes the protocol for an Intervention that aims at supporting healthy infant feeding practices to prevent childhood obesity in the first year.

 The only issue that is unclear to me is if the intervention intends to have an effect on the first year of life why does the eligibility criteria include parents with children up to 2 years of age, wouldn't it be better to have parents with children under one year of age?

 Everything else is clear and congratulations for this important work.

Is the study design appropriate for the research question?

Yes

Is the rationale for, and objectives of, the study clearly described?

Yes

Are sufficient details of the methods provided to allow replication by others?

Yes

Are the datasets clearly presented in a useable and accessible format?

Not applicable

Reviewer Expertise:

Intervention science around childhood and adolescent obesity prevention.

I confirm that I have read this submission and believe that I have an appropriate level of expertise to confirm that it is of an acceptable scientific standard.

# Reviewer report: Reviewer response for version 1

**DOI:** 10.21956/hrbopenres.15290.r42691

**Published:** 2024-11-06

**Authors:** Julie A Lanigan

**Affiliations:** 1 University College London, London, England, UK

The article provides a reasonable background to justify refinement to the CHErIsH protocol. I have only minor suggestions to make.

 Firstly, be clear that infants are aged up to 1 year. Beyond that 'child' is the appropriate descriptor.

 The expertise of the range of HCPs in infant and young child feeding is questionable. It is well-recognised that medical doctors and many HCPs are not well-educated in nutrition. Dietitians are the HCPs with the highest level of knowledge and expertise in the area of infant and young child feeding. Suggest this is acknowledged and this profession is fully utilised in the survey process.

 Consider the use of obesity as a descriptor applied to an infant. Excessive weight gain may be more appropriate here as infants and young children under the age of 2 cannot be classified as obese.This terminology may be upsetting for parents and caregivers.

Is the study design appropriate for the research question?

Yes

Is the rationale for, and objectives of, the study clearly described?

Yes

Are sufficient details of the methods provided to allow replication by others?

Yes

Are the datasets clearly presented in a useable and accessible format?

Not applicable

Reviewer Expertise:

Childhood obesity prevention and treatment. Infant and young child feeding. Childhood nutrition and relationships to short and long-term health.

I confirm that I have read this submission and believe that I have an appropriate level of expertise to confirm that it is of an acceptable scientific standard.
